# Supplementary material for: Cytokine ranking via mutual information algorithm correlates cytokine profiles with presenting disease severity in patients infected with SARS-CoV-2
Source: eLife. 2021 Jan 14;10:e64958. doi: 10.7554/eLife.64958 (PMC7872512; doi:10.7554/eLife.64958)
Supplement: Figure 1—source data 1. [file elife-64958-fig1-data1.docx]

**SOURCE DATA**

**Source Data Table 1. Mutual Algorithm Criteria Table**

**
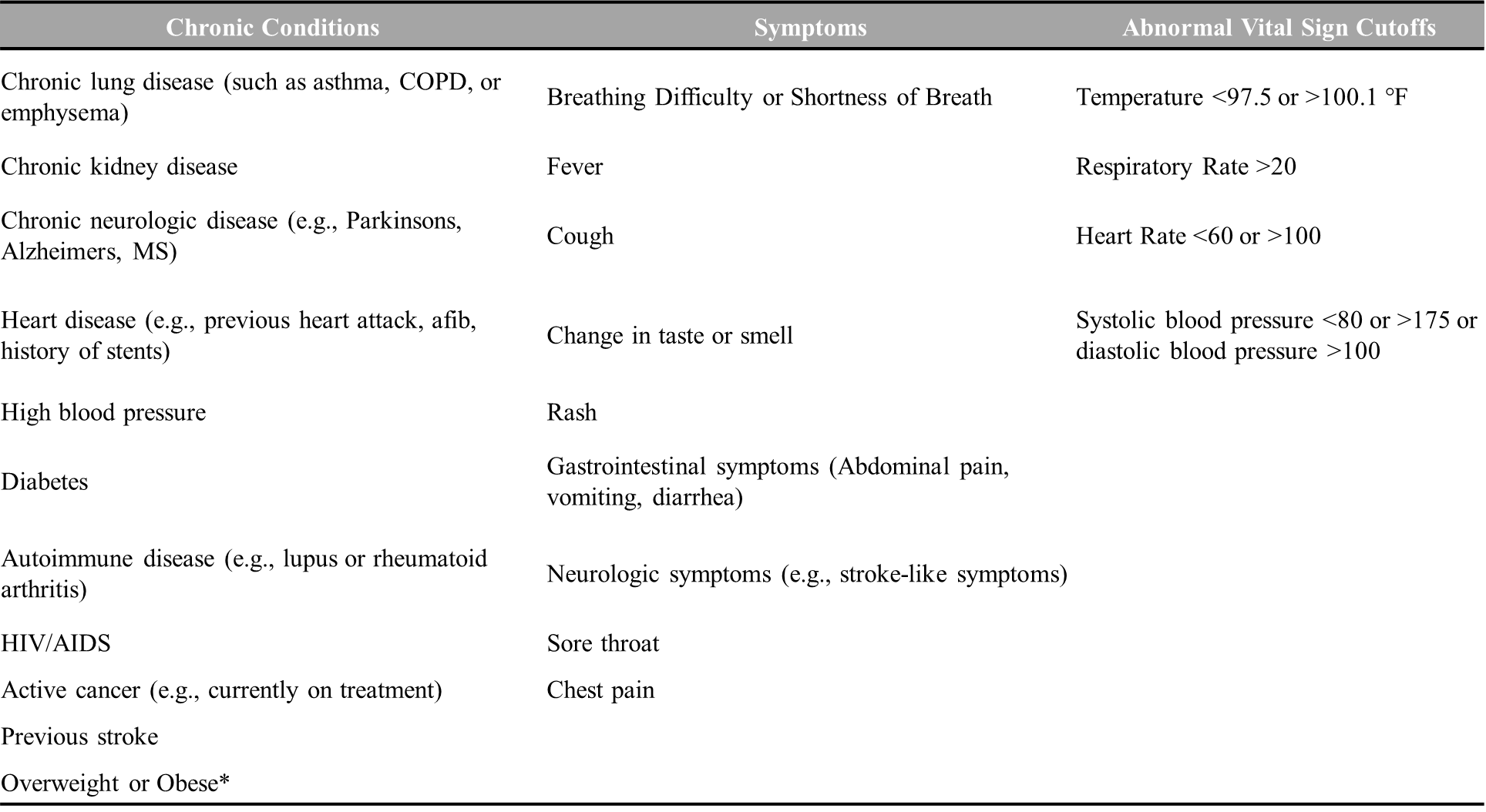
**

*documented obesity or overweight for height <99th percentile
