# Supplementary material for: Cytokine ranking via mutual information algorithm correlates cytokine profiles with presenting disease severity in patients infected with SARS-CoV-2
Source: eLife. 2021 Jan 14;10:e64958. doi: 10.7554/eLife.64958 (PMC7872512; doi:10.7554/eLife.64958)
Supplement: Figure 2—source data 1. [file elife-64958-fig2-data1.docx]

**SOURCE DATA**

**Source Data Table 2. Patient Information**

| Patient Information | | | | |
| --- | --- | --- | --- | --- |
| Sample ID | **Age** | **Sex** | **Race** | **COVID Severity Score** |
| 1 | 43 | Male | Black | 2 |
| 2 | 67 | Male | Black | 5 |
| 4 | 64 | Male | Hispanic or Latino | 3 |
| 5 | 73 | Male | White | 4 |
| 6 | 50 | Male | Other | 4 |
| 7 | 35 | Male | White | 2 |
| 8 | 57 | Male | Other | 5 |
| 11 | 61 | Male | Asian or Pacific Islander | 2 |
| 13 | 17 | Female | Unknown | 2 |
| 15 | 48 | Female | White | 1 |
| 16 | 25 | Male | Hispanic or Latino | 1 |
| 17 | 28 | Male | Other | 2 |
| 21 | 44 | Male | Hispanic or Latino | 2 |
| 23 | 51 | Male | Other | 1 |
| 24 | 30 | Male | Black | 1 |
| 26 | 35 | Female | Other | 2 |
| 29 | 48 | Male | Hispanic or Latino | 4 |
| 31 | 55 | Male | Hispanic or Latino | 1 |
| 33 | 30 | Female | White | 2 |
| 35 | 33 | Male | Hispanic or Latino | 5 |
| 42 | 71 | Female | White | 5 |
| 44 | 54 | Female | Asian or Pacific Islander | 2 |
| 45 | 30 | Male | Other | 1 |
| 46 | 74 | Female | Hispanic or Latino | 2 |
| 48 | 53 | Female | Hispanic or Latino | 4 |
| 49 | 85 | Female | Hispanic or Latino | 3 |
| 53 | 80 | Male | Hispanic or Latino | 4 |
| 62 | 77 | Male | Hispanic or Latino | 3 |
| 63 | 42 | Female | Hispanic or Latino | 3 |
| 69 | 82 | Male | Black | 1 |
| 70 | 23 | Male | Hispanic or Latino | 2 |
| 71 | 23 | Female | Black | 1 |
| 76 | 20 | Male | Hispanic or Latino | 1 |
| 77 | 21 | Male | Hispanic or Latino | 2 |
| 78 | 72 | Male | Hispanic or Latino | 3 |
| 79 | 75 | Male | White | 1 |
| 82 | 91 | Female | White | 4 |
| 84 | 41 | Female | Hispanic or Latino | 2 |
| 94 | 50 | Male | Hispanic or Latino | 3 |
| 101 | 19 | Male | Hispanic or Latino | 2 |
| 102 | 63 | Female | Hispanic or Latino | 4 |
| 103 | 73 | Male | White | 5 |
| 115 | 35 | Female | White | 1 |
| 118 | 22 | Female | Hispanic or Latino | 3 |
| 122 | 51 | Male | Hispanic or Latino | 5 |
| 126 | 27 | Female | Asian or Pacific Islander | 2 |
| 127 | 44 | Female | Black | 1 |
| 130 | 68 | Female | White | 4 |
| P699219 | 50 | Female | White | 0 |
| P699245 | 46 | Female | White | 0 |
| P699274 | 34 | Male | White | 0 |
| P699291 | 61 | Female | White | 0 |
| P699398 | 50 | Male | Asian | 0 |
| P757865 | 70 | Male | White | 0 |
| P761842 | 27 | Female | White | 0 |
| P761936 | 32 | Male | Asian | 0 |
| P761951 | 70 | Female | White | 0 |
| P762878 | 20 | Female | White | 0 |
| P762884 | 56 | Female | White | 0 |
| P762937 | 56 | Male | White | 0 |
| P762976 | 62 | Male | White | 0 |
| P762995 | 76 | Male | White | 0 |
| P763007 | 30 | Female | White | 0 |
| P763051 | 62 | Male | Hispanic | 0 |
| P763071 | 33 | Female | Hispanic | 0 |
| P763072 | 40 | Male | White | 0 |
| P763137 | 57 | Female | White | 0 |
